# Fluid strategies and outcomes in patients with acute respiratory distress syndrome, systemic inflammatory response syndrome and sepsis: a protocol for a systematic review and meta-analysis

**DOI:** 10.1186/s13643-015-0150-z

**Published:** 2015-11-12

**Authors:** Jonathan A. Silversides, Andrew J. Ferguson, Daniel F. McAuley, Bronagh Blackwood, John C. Marshall, Eddy Fan

**Affiliations:** Centre for Infection and Immunity, School of Medicine, Dentistry and Biomedical Sciences, Queen’s University Belfast, 97 Lisburn Road, Belfast, BT9 7BL UK; Critical Care Services, Belfast Health and Social Care Trust, Belfast, UK; Department of Anaesthetics and Intensive Care, Southern Health and Social Care Trust, Craigavon Area Hospital, 68 Lurgan Road, Portadown, BT63 5QQ UK; Interdepartmental Division of Critical Care, University of Toronto, Toronto General Hospital, 585 University Avenue, PMB 11-123, Toronto, Ontario M5G 2N2 Canada; Critical Care Medicine, St. Michael’s Hospital, Toronto, 30 Bond Street, Bond 4-014, Toronto, Ontario M5B 1W8 Canada; Critical Care Medicine, University Health Network and Mount Sinai Hospitals, Toronto, Canada

**Keywords:** Fluid therapy, Sepsis, Sepsis syndrome, Systemic inflammatory response syndrome, Respiratory distress syndrome, Adult, Deresuscitation, Ultrafiltration, Diuretics, Critical illness

## Abstract

**Background:**

Fluid administration to critically ill patients remains the subject of considerable controversy. While intravenous fluid given for resuscitation may be life-saving, a positive fluid balance over time is associated with worse outcomes in critical illness. The aim of this systematic review is to summarise the existing evidence regarding the relationship between fluid administration or balance and clinically important patient outcomes in critical illness.

**Methods:**

We will search Medline, EMBASE, the Cochrane Central Register of Controlled Trials from 1980 to the present and key conference proceedings from 2009 to the present. We will include studies of critically ill adults and children with acute respiratory distress syndrome (ARDS), sepsis and systemic inflammatory response syndrome (SIRS). We will include randomised controlled trials comparing two or more fluid regimens of different volumes of fluid and observational studies reporting the relationship between volume of fluid administered or fluid balance and outcomes including mortality, lengths of intensive care unit and hospital stay and organ dysfunction. Two independent reviewers will assess articles for eligibility, data extraction and quality appraisal. We will conduct a narrative and/or meta-analysis as appropriate.

**Discussion:**

While fluid management has been extensively studied and discussed in the critical care literature, no systematic review has attempted to summarise the evidence for post-resuscitation fluid strategies in critical illness. Results of the proposed systematic review will inform practice and the design of future clinical trials.

**Systematic review registration:**

PROSPERO CRD42013005608. (http://www.crd.york.ac.uk/PROSPERO/)

**Electronic supplementary material:**

The online version of this article (doi:10.1186/s13643-015-0150-z) contains supplementary material, which is available to authorized users.

## Background

### Description of the condition

Despite a vast range of underlying aetiologies for critical illness, patients who are critically ill have numerous features in common and often share a similar approach to management. For this review, however, we have selected critical illnesses for which clear definitions exist—acute respiratory distress syndrome (ARDS), sepsis and systemic inflammatory response syndrome (SIRS)—since ‘critical illness’ is a poorly defined entity. Each of these terms represents a common syndrome found in critically ill patients with a range of underlying aetiologies. The majority of patients who are admitted to an intensive care unit will have one or more of these syndromes. ARDS encompasses the older term ‘acute lung injury (ALI)’ and refers to an acute inflammatory process involving both lungs resulting in hypoxaemia and low lung compliance. ARDS occurs in around 14 % of patients admitted to intensive care units (ICUs) [1] and is defined most recently by the Berlin consensus criteria [2]. SIRS is a general term describing a constellation of features which are consistent with generalised inflammation and which may result from a range of insults including infection, major trauma, burns, pancreatitis and brain injury. SIRS has been reported to occur in up to 98 % of patients in the intensive care environment [3]. Sepsis is defined as infection with SIRS and is found in approximately 37 % of ICU patients [4]. By using as our study population patients with each of these syndromes, we aim to set clear boundaries for inclusion and exclusion of studies while maximising the generalisability of our findings to critically ill patients as far as possible.

### Description of the intervention

Intravenous fluid administration is one of the most common interventions in critical care. In patients with hypovolaemia or pathological vasodilation from states such as sepsis, it is widely held that early and rapid administration of intravenous fluid to restore circulating blood volume and blood flow to tissues is life-saving, although that view has recently been challenged [5]. The volume of intravenous fluid which may be given is highly variable and is dependent on the endpoints used to assess tissue perfusion, among other factors [6].

Despite large volumes of literature on the subject, the optimal targets for fluid resuscitation remain unclear, while fluid is also administered to critically ill patients as maintenance fluid, drug diluent, nutrition and blood products. Moreover, in critical illness states, sodium and water retention is common due to prevailing endocrine influences and to the frequent occurrence of acute kidney injury. The combination of fluid administration and limited excretion typically leads to a positive fluid balance of many litres over a period of days, which is compounded by fluid leak from capillaries into the interstitium to produce oedema of peripheries, lungs, and other organs [7].

The paradox is that while fluid resuscitation early in critical illness is thought to be life-saving, a positive cumulative fluid balance is associated with mortality in patients with septic shock [4, 8] and in subgroups with acute kidney injury (AKI) [9–11] or ARDS [12–14]. In contrast, in the setting of ARDS, diuretic use [12] and restrictive fluid management are associated with lower mortality and faster liberation from mechanical ventilation, respectively [14]. At present, there is equipoise as to whether the association between fluid accumulation and poor outcomes represents a causal relationship, or is simply a marker of more severely ill patients who require more fluid resuscitation, are oliguric, or who fail to tolerate the use of diuretics or ultrafiltration to remove accumulated fluid, a strategy known as deresuscitation.

### How the intervention might work

As a result of these seemingly conflicting findings, a multi-phasic approach to fluid therapy has been proposed. It is postulated that while fluid resuscitation early in the course of critical illness to optimise cardiac output and tissue perfusion is beneficial, a conservative fluid management strategy or active deresuscitation in the later phase of critical illness may improve organ function and survival by minimising the harmful effects of tissue oedema [15]. However, a post hoc analysis of ARDS survivors from the Fluids and Catheter Treatment Trial [14] found a conservative fluid management strategy to be associated with long-term cognitive impairment [16]. Thus, the risks and benefits of a conservative fluid strategy, along with the applicability of this finding to patients without ARDS, remain uncertain.

### Why it is important to do this review

Fluid management is a key element of the management of patients who are critically ill. Optimisation of oxygen delivery to tissues requires fluid administration, while a growing body of observational literature points to a consistent association between fluid overload and worse patient outcomes. Developing our understanding of the benefits and harms of fluid therapy is a fundamental question of major relevance to the practice of critical care medicine.

### Review question

In adult and paediatric patients with sepsis, SIRS or ARDS who are not in the resuscitation phase of critical illness, is a more restrictive fluid regimen/active deresuscitation strategy associated with an improvement in clinically important outcomes?

### Sub-questions

1. What criteria are used to judge suitability for fluid restriction or deresuscitation?
2. What methods are used to restrict fluid or deresuscitate?
3. What contra-indications to deresuscitation are applied?

## Methods

### Types of studies

We will include:

1. All clinical trials (randomised or quasi-randomised) comparing two or more fluid regimens in which different volumes of fluid are administered or different fluid balance is achieved, regardless of techniques used to guide fluid therapy
2. Observational studies in which fluid management is a major focus of the study and in which the study reports the relationship between the volume of fluid administered or fluid balance and one or more of the outcomes above

We will exclude case series or reports, observational studies in which the total number of subjects is less than 50, and studies subject to post-publication investigation or retraction.

### Types of participants

We will include studies of adult and paediatric patients. Since ‘critical illness’ is a poorly defined entity, we will include critical illnesses for which widely accepted definitions exist: ARDS, sepsis and SIRS in this review. We will exclude studies involving animals, or in which the patient population is made up predominantly of neonates, post-cardiac surgery or heart failure patients or perioperative patients.

### Types of intervention(s) and comparators

Studies will be included if the intervention involved minimisation of fluid intake or active deresuscitation using diuretics or ultrafiltration and if the comparator was standard care or liberal fluid strategy.

We will exclude studies in which fluid management is only one aspect of a haemodynamic intervention strategy or in which the main comparison is between two or more different types of fluid.

### Types of outcome measures

1. Primary outcome:
  Mortality (all cause, at the most protracted time point reported to a maximum of 90 days)
2. Secondary outcomes:
  Length of ICU stay, length of hospital stay
  Organ dysfunction (multiple organ dysfunction score (MODS), sequential organ failure score (SOFA) or simplified acute physiology score (SAPS II))
  Renal dysfunction (AKI incidence, renal replacement therapy use)
  Respiratory dysfunction (incidence of new ARDS, ventilator-free days)
  Cognitive function (author-defined)
  Long-term mortality (>90 days)

### Search strategy

We will search the following databases for relevant studies from 1980 onwards with no language constraints: Medline, EMBASE, and Cochrane Central Register of Controlled Trials (CENTRAL). In addition, we will undertake a manual search of indexed abstracts from the following key conference proceedings from 2009 to the present: the American Thoracic Society, Society of Critical Care Medicine, and European Society of Intensive Care Medicine Annual Congresses and the International Symposium on Intensive Care and Emergency Medicine.

Key search terms will include the following: fluid therapy, diuretics, furosemide, ultrafiltration, water-electrolyte balance, systemic inflammatory response syndrome, sepsis, acute lung injury, respiratory distress syndrome and adult. Our Medline search strategy (Additional file 1) will be adapted for searches in other databases.

### Citation management and screening

Citations will be stored using Microsoft Excel software (Microsoft Corporation, Redmond, WA), and duplicates will be removed. Studies will be screened initially according to title and abstract by two authors independently, and those not meeting the criteria will be discarded. Disagreements will be resolved by discussion and referral to a third author if necessary.

After this initial stage, the full text of all remaining studies will be reviewed by two authors independently for inclusion or exclusion in the final study. As before, disagreements will be resolved by discussion and referral to a third author if necessary.

### Data abstraction

For interventional studies, we will record information on the type and setting of the study, number of subjects, patient characteristics, nature of the intervention(s) in each group and outcomes as described above. After piloting, data will be extracted independently by two authors using standardised report forms (Additional file 2). Adjudication by a third author will be used if necessary.

For observational studies, a minimal dataset will include study details, patient characteristics, description of the intervention(s), main outcomes and adverse events. Efforts will be made to contact the authors of primary studies to provide missing data where necessary.

### Assessment of risk of bias

The quality of included studies will be independently assessed by two authors and verified by a third if necessary. Study quality of RCTs will be assessed using the domain-based evaluation recommended by the Cochrane Collaboration [17]. The domains include:

1. Random sequence generation
2. Allocation concealment
3. Blinding
4. Incomplete outcome data
5. Selective reporting
6. Other bias

For each domain, we will assign a judgement regarding the risk of bias as high, low or unclear [17]. We will attempt to contact the trial corresponding author for clarification when insufficient detail is reported to assess risk of bias. Once we have consensus on the quality assessment of the six domains for eligible studies, we will assign them to the following categories:

1. Low risk of bias: describes studies for which all domains are scored as ‘yes’
2. Moderate risk of bias: describes studies for which one or more domains are scored as unclear or one domain is scored as ‘no’
3. High risk of bias: describes studies for which more than one domain is scored as ‘no’

We will construct a ‘Risk of Bias’ table to present the results. We will use the assessment of risk of bias to perform sensitivity analyses based on methodological quality as necessary.

For observational studies, the Newcastle-Ottawa Scale will be used to assess study quality. Domains used to judge the quality of cohort studies include:

1. Representativeness of the exposed cohort
2. Selection of the non-exposed cohort
3. Ascertainment of exposure
4. Demonstration that outcome of interest was not present at the start of the study
5. Comparability of cohorts on the basis of the design or analysis
6. Assessment of outcome

Case-control studies will be assessed using the appropriate version of this scale. Reporting of all observational studies will include this assessment of study quality.

### Data synthesis

If two or more randomised controlled trials are available for an outcome, their results will be combined in a meta-analysis using Review Manager (RevMan 5.3) software (Nordic Cochrane Centre, Cochrane Collaboration) on an intention-to-treat basis if appropriate to do so. We will not attempt meta-analysis of observational studies. If heterogeneity is very high (i.e. >90 %) and is unexplained by pre-planned subgroup analysis, we will report results narratively. If there are data from only one study for an outcome, the results will be reported narratively. For continuous data, we will use the inverse variance method, while the Mantel-Haenszel method will be employed for dichotomous data. For key outcomes, we will evaluate our confidence in the body of evidence according to the GRADE scale ([18]).

### Measures of effect for dichotomous data

Where possible, the treatment effect for dichotomous outcomes will be expressed as odds ratios with 95 % confidence intervals. If time-to-event data is available, we will use hazard ratios rather than risk ratios. We will use the Peto method to estimate the odds ratios for rare outcomes.

### Measures of effect for continuous data

The treatment effect for continuous outcomes will be expressed as mean difference with 95 % confidence intervals. Where different scales are used, standardised mean difference (SMD) with 95 % confidence intervals will be used to express treatment effect.

### Measures of effect for ordinal data

We will analyse longer ordinal scales as continuous data, while shorter ordinal scales will be abbreviated to dichotomous data. Treatment effects will then be assessed as described for each type of data.

### Unit of analysis issues

Individual participants in each trial arm will comprise the unit of analysis. We anticipate that all trials included in the meta-analysis will have a parallel group design, and thus, no adjustment will be necessary for crossover or clustering.

### Missing data

Where possible, we will contact trial authors to request missing data.

### Assessment of heterogeneity

We will assess qualitative heterogeneity of the included studies and only combine studies whose participants are sufficiently similar, such that combining data across studies leads to a meaningful result. Statistical heterogeneity will be informally evaluated from forest plots of the study estimates and more formally using the chi^2^ test (*P* value <0.1, significant heterogeneity) and *I*^2^ statistic (*I*^2^ > 50 % = significant heterogeneity) [17].

### Assessment of reporting biases

If a sufficient number of studies is identified (*n* > 10), we will investigate reporting biases through the use of a funnel plot.

### Sensitivity and subgroup analysis

If appropriate, we will investigate the influence of methodological quality on results by examining the effect of excluding studies at high or unclear risk of bias, and if necessary, we will undertake sensitivity analysis to assess the effect of excluding individual studies.

If sufficient studies are available, we will undertake subgroup analyses for adults, children, all patients with ALI or ARDS and all patients with sepsis, SIRS or septic shock.

### Standards

Reporting will conform to Preferred Reporting Items for Systematic Reviews and Meta Analyses (PRISMA) standards (Additional file 3) [19]. This systematic review has been registered with PROSPERO, an international prospective register of systematic reviews (http://www.crd.york.ac.uk/PROSPERO/).

## Discussion

While fluid management has been extensively studied and discussed in the critical care literature, no systematic review to date has attempted to summarise the evidence for a greater or lesser fluid volume or balance at different time points in critical illness, and this remains an area of debate among clinicians and researchers. It is hoped that in summarising the current evidence, this systematic review will inform practice and future trial design.

## Additional files

Additional file 1:
**Medline search strategy.** Modified versions of this strategy will be used for other databases. Additional file 2:
**Data abstraction form for randomised controlled trials.** A modified version of this form will be used for observational studies. Additional file 3:
**Checklist of Preferred Reporting Items for Systematic Reviews and Meta-analysis Protocols (PRISMA-P).** Reporting guideline for systematic review protocols.

## Abbreviations

AKI: acute kidney injury
ALI: acute lung injury
ARDS: acute respiratory distress syndrome
CENTRAL: Cochrane Central Register of Controlled Trials
ICU: intensive care unit
MODS: multiple organ dysfunction score
SAPS II: simplified acute physiology score
SIRS: systemic inflammatory response syndrome
SOFA: sequential organ failure score
