# Supplementary material for: Fluid strategies and outcomes in patients with acute respiratory distress syndrome, systemic inflammatory response syndrome and sepsis: a protocol for a systematic review and meta-analysis
Source: Syst Rev. 2015 Nov 12;4:162. doi: 10.1186/s13643-015-0150-z (PMC4643493; doi:10.1186/s13643-015-0150-z)
Supplement: Additional file 1: — Medline search strategy. Modified versions of this strategy will be used for other databases. [file 13643_2015_150_MOESM1_ESM.docx]

Database: Ovid MEDLINE(R) <1946 to June Week 4 2013>

Search Strategy:

--------------------------------------------------------------------------------

1 exp fluid therapy/ (14133)

2 infusions, parenteral/ (25202)

3 infusions, intravenous/ (49278)

4 (fluid* adj2 therap*).tw. (2531)

5 (infus* adj2 intraven*).tw. (32271)

6 (infus* adj2 parenter*).tw. (452)

7 (infus* adj2 intraoss*).tw. (278)

8 (intraven* adj2 drip*).tw. (1366)

9 (iv adj2 bolus).tw. (5300)

10 (intraven* adj2 bolus).tw. (7338)

11 (fluid* adj2 replac*).tw. (2062)

12 (fluid* adj2 restor*).tw. (265)

13 (fluid* adj2 administ*).tw. (2563)

14 (fluid* adj2 manag*).tw. (1479)

15 (volum* adj2 restor*).tw. (642)

16 (fluid* adj2 resuscitat*).tw. (3469)

17 (fluid* adj2 restrict*).tw. (1376)

18 exp diuretics/ (70728)

19 furosemide/ (11116)

20 furosemid*.tw. (9962)

21 frusemid*.tw. (1351)

22 ultrafiltrat*.tw. (12800)

23 catheterization, swan-ganz/ (2126)

24 (catheter* adj2 swan-ganz).tw. (1823)

25 (catheter* adj2 swanganz).tw. (2)

26 (pulmonary adj3 catheter*).tw. (4626)

27 exp water-electrolyte balance/ (28416)

28 (water-electrolyte adj2 balanc*).tw. (470)

29 (water-electrolyte adj2 imbalanc*).tw. (101)

30 (fluid* adj2 balanc*).tw. (3562)

31 (fluid* adj2 imbalanc*).tw. (177)

32 (water adj2 balanc*).tw. (3395)

33 (water adj2 imbalanc*).tw. (206)

34 extravascular lung water/ (989)

35 (extravascul* adj2 water).tw. (1399)

36 thermodilution/ (2327)

37 thermodilut*.tw. (3931)

38 hemodynamics/ (118846)

39 hemodynam*.tw. (108689)

40 (lithium adj2 dilut*).tw. (81)

41 (pulse adj2 contour$ analy*).tw. (270)

42 (#esophag* adj2 doppler).tw. (148)

43 picco.tw. (238)

44 (stroke adj2 volume adj2 varia*).tw. (277)

45 (stroke adj2 volume adj2 change$).tw. (490)

46 (stroke adj2 volume adj2 adjust*).tw. (17)

47 (stroke adj2 volume adj2 alter*).tw. (42)

48 (pulse adj2 pressure adj2 varia*).tw. (302)

49 (pulse adj2 pressure adj2 change$).tw. (178)

50 (pulse adj2 pressure adj2 adjust*).tw. (34)

51 (pulse adj2 pressure adj2 alter*).tw. (24)

52 pulmonary wedge pressure/ (4746)

53 (pulmonary adj2 wedge adj2 pressur*).tw. (5360)

54 exp colloids/ (92282)

55 colloid*.tw. (28000)

56 hetastarch/ (2403)

57 hetastarch*.tw. (430)

58 sodium chloride/ (50027)

59 sodium chloride.tw. (11984)

60 crystalloid*.tw. (4680)

61 (saline adj2 solution$).tw. (12921)

62 saline solution, hypertonic/ (4898)

63 diuret*.mp. (44136)

64 Blood Pressure/ (237740)

65 haemodynamic*.tw. (24747)

66 (extravascul* adj2 fluid*).tw. (428)

67 (infus* adj2 fluid*).tw. (1988)

68 (lung adj2 wedge adj2 pressur*).tw. (1)

69 or/1-68 (788631)

70 exp systemic inflammatory response syndrome/ (95029)

71 systemic inflammatory response syndrome.tw. (2727)

72 exp bacteremia/ (21311)

73 endotoxemia/ (3429)

74 endotoxemi*.tw. (6272)

75 bacteremi*.tw. (17738)

76 sepsis.tw. (59877)

77 septic.tw. (34541)

78 hemorrhagic septicemia/ (153)

79 (hemorrhag* adj2 septic*).tw. (530)

80 shock, septic/ (18180)

81 exp shock/ (58815)

82 (septic adj2 shock).tw. (13317)

83 Respiratory Distress Syndrome, Adult/ (14813)

84 (adult adj2 respiratory distress syndrome$).tw. (4061)

85 vasoplegia/ (40)

86 vasoplegia$.tw. (114)

87 (acute adj2 ill*).tw. (6474)

88 (acute adj2 injur*).tw. (23750)

89 Acute Lung Injury/ (3185)

90 (acute adj2 lung$ adj2 injur*).tw. (8943)

91 multiple organ failure/ (8509)

92 (multi* adj2 organ$ adj2 fail*).tw. (7419)

93 (acute adj2 respira* adj2 fail*).tw. (4813)

94 Sepsis/ (42656)

95 sirs.tw. (2985)

96 septicaemi*.tw. (5559)

97 septicemi*.tw. (11147)

98 bacteraemi*.tw. (4417)

99 endotoxaemi*.tw. (968)

100 (acute adj2 respiratory distress syndrome$).tw. (6806)

101 (lung$ adj2 shock).tw. (537)

102 vasoplegi*.tw. (189)

103 (multi* adj2 organ$ adj2 dysfunction).tw. (2770)

104 (bacter* adj2 shock).tw. (688)

105 (endotox* adj2 shock).tw. (3957)

106 (toxi* adj2 shock).tw. (3978)

107 (shock adj2 syndrom*).tw. (4905)

108 Capillary Leak Syndrome/ (469)

109 (capillar* adj2 leak*).tw. (1380)

110 (blood* adj2 poison*).tw. (135)

111 pyohemia*.tw. (7)

112 pyemia*.tw. (44)

113 pyaemia*.tw. (39)

114 or/70-113 (246875)

115 69 and 114 (30240)

116 115 not (animals not humans).hw. (19424)

***************************
