# Supplementary material for: Fluid strategies and outcomes in patients with acute respiratory distress syndrome, systemic inflammatory response syndrome and sepsis: a protocol for a systematic review and meta-analysis
Source: Syst Rev. 2015 Nov 12;4:162. doi: 10.1186/s13643-015-0150-z (PMC4643493; doi:10.1186/s13643-015-0150-z)
Supplement: Additional file 2: — Data abstraction form for randomised controlled trials. A modified version of this form will be used for observational studies. [file 13643_2015_150_MOESM2_ESM.docx]

RADAR SYSTEMATIC REVIEW DATA EXTRACTION FORM (RCTs)

| **GENERAL STUDY INFO** | | | | | | | | | | | | | | | | | | | | | |
| --- | --- | --- | --- | --- | --- | --- | --- | --- | --- | --- | --- | --- | --- | --- | --- | --- | --- | --- | --- | --- | --- |
| **Ref ID** | | | **Title** | | | | | | | | | | | | | | **Authors** | | | | |
|  | | |  | | | | | | | | | | | | | |  | | | | |
| **Year** | | | **Startpage** | | **Endpage** | | | **Vol** | | | **Journal** | | | | | |  | | | | |
|  | | |  | |  | | |  | | |  | | | | | |  |  |  |  |  |
| **Include on full text review:** | | | | | **Y** |  | | **N** | |  | | **Exclusion reason** | | | | |  | | | | |
| **NOTES** | | | | | | | | | | | | | | | | | | | | | |
| **STUDY DESIGN** | | | | | | | | | | | | | | | | | | | | | |
| **RCT** | |  | | **CLUSTER-RCT** | | | |  | **QUASI-RCT** | | | |  | | **OTHER** |  | | **Single centre** |  | **Multi-centre** |  |
| **PARTICIPANTS** | | | | | | | | | | | | | | | | | | | | | |
| **Inclusion criteria:** | | | | | | | | | | | | | | | | | | | | | |
| **Exclusion criteria:** | | | | | | | | | | | | | | | | | | | | | |
| **Adults:** | **Y / N** | | | **Children:** | | | **Y / N** | | **Country** | | | |  | | | | **Community/Academic** | |  | | |
| **More fluid group** | | | | | | | | | | | | | | **Less fluid group** | | | | | | | |

| **N=** |  | | | **Disease process**  **(eg ARDS, SIRS)** | | | |  | | | **N=** |  | | **Disease process**  **(eg ARDS, SIRS)** | | |  | |
| --- | --- | --- | --- | --- | --- | --- | --- | --- | --- | --- | --- | --- | --- | --- | --- | --- | --- | --- |
| **Diagnostic criteria:** | | | | |  | | | | | | **Diagnostic criteria:** | | | | | |  | |
| **Time from ICU admission to enrollment:** | | | | |  | | | | | | **Time from ICU admission to enrollment:** | | | | | |  | |
| **Age:** | **Mean** | | | | **SD** | | **Median** | | | **IQR** | **Mean** | | | **SD** | | | **Median** | **IQR** |
|  |  | | | |  | |  | | |  |  | | |  | | |  |  |
| **Male sex:** | | **N** | | |  | **%** |  | |  | | **Male sex:** | | **N** |  | **%** |  |  | |
| **Severity of illness scoring system used:**  **(eg APACHE 2, MODS, SAPS 2)** | | | | | | | | |  | | **Severity of illness scoring system used:**  **(eg APACHE 2, MODS, SAPS 2)** | | | | | |  | |
| **Severity score:** | | | **Mean** | | **SD** | | **Median** | | | **IQR** | **Mean** | | | **SD** | | | **Median** | **IQR** |
|  | | |  | |  | |  | | |  |  | | |  | | |  |  |
| **NOTES:** | | | | | | | | | | | | | | | | | | |

| **INTERVENTION DETAILS:**  **PLEASE RECORD UNITS OF MEASUREMENT FOR ALL VARIABLES** | | | | | | | |
| --- | --- | --- | --- | --- | --- | --- | --- |
| **More fluid group** | | | | **Less fluid group** | | | |
| **Description of fluid management strategy:**  **(including monitoring, fluid responsiveness testing, fluid dose, diuretic dose)** | | | | **Description of fluid management strategy:**  **(including monitoring, fluid responsiveness testing, fluid dose, diuretic dose)** | | | |
| **Fluid type (crystalloid, colloid, other):** | | | | **Fluid type (crystalloid, colloid, other):** | | | |
| **Fluid balance (24 hours):** | | | | | | | |
| **Mean** | **SD** | **Median** | **IQR** | **Mean** | **SD** | **Median** | **IQR** |
|  |  |  |  |  |  |  |  |
| **Fluid administration (24 hours):** | | | | | | | |
| **Mean** | **SD** | **Median** | **IQR** | **Mean** | **SD** | **Median** | **IQR** |
|  |  |  |  |  |  |  |  |
| **Fluid balance (48 hours / 2 days):** | | | | | | | |
| **Mean** | **SD** | **Median** | **IQR** | **Mean** | **SD** | **Median** | **IQR** |
|  |  |  |  |  |  |  |  |
| **Fluid administration (48 hours / 2 days):** | | | | | | | |
| **Mean** | **SD** | **Median** | **IQR** | **Mean** | **SD** | **Median** | **IQR** |
|  |  |  |  |  |  |  |  |

| **Fluid balance (72 hours / 3 days):** | | | | | | | | | |
| --- | --- | --- | --- | --- | --- | --- | --- | --- | --- |
| **Mean** | **SD** | **Median** | **IQR** | | **Mean** | | **SD** | **Median** | **IQR** |
|  |  |  |  | |  | |  |  |  |
| **Fluid administration (72 hours / 3 days):** | | | | | | | | | |
| **Mean** | **SD** | **Median** | **IQR** | | **Mean** | | **SD** | **Median** | **IQR** |
|  |  |  |  | |  | |  |  |  |
| **Fluid balance (7 days):** | | | | | | | | | |
| **Mean** | **SD** | **Median** | | **IQR** | **Mean** | | **SD** | **Median** | **IQR** |
|  |  |  | |  |  | |  |  |  |
| **Fluid administration (7 days):** | | | | | | | | | |
| **Mean** | **SD** | **Median** | | **IQR** | | **Mean** | **SD** | **Median** | **IQR** |
|  |  |  | |  | |  |  |  |  |
| **Fluid balance (other point):** | | | | | | | | | |
| **Mean** | **SD** | **Median** | | **IQR** | **Mean** | | **SD** | **Median** | **IQR** |
|  |  |  | |  |  | |  |  |  |
| **Fluid administration (other point):** | | | | | | | | | |
| **Mean** | **SD** | **Median** | | **IQR** | | **Mean** | **SD** | **Median** | **IQR** |
|  |  |  | |  | |  |  |  |  |
| **Fluid balance (last recorded point):** | | | | | | | | | |
| **Mean** | **SD** | **Median** | | **IQR** | **Mean** | | **SD** | **Median** | **IQR** |
|  |  |  | |  |  | |  |  |  |
| **Fluid administration (last recorded point):** | | | | | | | | | |
| **Mean** | **SD** | **Median** | | **IQR** | | **Mean** | **SD** | **Median** | **IQR** |
|  |  |  | |  | |  |  |  |  |

| **OUTCOMES** | | | | | | | | | | | | | | | | | | |
| --- | --- | --- | --- | --- | --- | --- | --- | --- | --- | --- | --- | --- | --- | --- | --- | --- | --- | --- |
| **More fluid group** | | | | | | | | **Less fluid group** | | | | | | | | | | |
| **ICU Mortality** | | | **N** |  | **of** | |  | **N** | |  | | **of** | | |  | |  | |
| **Hospital Mortality** | | | **N** |  | **of** | |  | **N** | |  | | **of** | | |  | |  | |
| **Day 28 Mortality** | | | **N** |  | **of** | |  | **N** | |  | | **of** | | |  | |  | |
| **Other Mortality timepoint (specify):** | | | **N** |  | **of** | |  | **N** | |  | | **of** | | |  | |  | |
| **More fluid group** | | | | | | | | **Less fluid group** | | | | | | | | | | |
| **AKI** | | | **N**  **N** |  | | **of** |  | **N** | | |  | | **of** | | |  | **AKI Definition used:** | |
| **RRT** | | | **N** |  | | **of** |  | **N** | | |  | | **of** | | |  |  | |
| **ARDS (new onset)** | | | **N** |  | | **of** |  | **N** | | |  | | **of** | | |  | **ARDS Definition used:** | |
| **Cognitive function (describe measures used, scales, and timing)** | | | | | | | | | | | | | | | | | | |
|  | | | | | | | |  | | | | | | | | | | |
| **Length of ICU stay** | | | | | | | | | | | | | | | | | | |
| **Mean** | **SD** | **Median** | | | **IQR** | | | **Mean** | **SD** | | | | | **Median** | | | | **IQR** |
|  |  |  | | |  | | |  |  | | | | |  | | | |  |
| **Ventilator free days** | | | | | | | | | | | | | | | | | | |
| **Mean** | **SD** | **Median** | | | **IQR** | | | **Mean** | **SD** | | | | | **Median** | | | | **IQR** |
|  |  |  | | |  | | |  |  | | | | |  | | | |  |
| **Length of hospital stay** | | | | | | | | | | | | | | | | | | |
| **Mean** | **SD** | **Median** | | | **IQR** | | | **Mean** | **SD** | | | | | **Median** | | | | **IQR** |
|  |  |  | | |  | | |  |  | | | | |  | | | |  |
| **Severity of illness (specify scale / change or absolute value / time point relative to intervention):** | | | | | | | | | | | | | | | | | | |
| **Mean** | **SD** | **Median** | | | **IQR** | | | **Mean** | **SD** | | | | | **Median** | | | | **IQR** |
|  |  |  | | |  | | |  |  | | | | |  | | | |  |
| **Other Outcomes / Notes** | | | | | | | |  | | | | | | | | | | |
|  | | | | | | | |  | | | | | | | | | | |

| **RISK OF BIAS MEASURES – PLEASE REFER TO COCHRANE RISK OF BIAS TABLE FOR ADDITIONAL DETAILS** | | | | | | | | | | |
| --- | --- | --- | --- | --- | --- | --- | --- | --- | --- | --- |
| **Recruitment period** | | | |  | | | | | | |
| **Intention to treat analysis** | **Y** |  | | **N** | | |  | | | |
| **SEQUENCE GENERATION** | | | | | | | | | | |
| **Description of method used for sequence generation:** | | | | | | | | | | |
| **Was the allocation sequence adequately generated to avoid selection bias?** | | | **Y** |  | **N** | |  | **Unclear** |  |  |
| **ALLOCATION CONCEALMENT** | | | | | | | | | | |
| **Description of methods used to conceal allocation to group:** | | | | | | | | | | |
| **Was the allocation adequately concealed to prevent selection bias?** | | | **Y** |  | **N** | |  | **Unclear** |  |  |
| **BLINDING of PARTICIPANTS / PERSONNEL** | | | | | | | | | | |
| **Description of measures used to prevent study participants and personnel from knowledge of the intervention group assigned, and effectiveness of these measures, if known:** | | | | | | | | | | |
| **Was knowledge of the allocated intervention by participants and personnel adequately prevented during the study?** | | | **Y** |  | **N** | |  | **Unclear** |  |  |
| **BLINDING OF OUTCOME ASSESSMENT** | | | | | | | | | | |
| **Description of any measures used to prevent knowledge of the assigned intervention by the outcome assessors and effectiveness, if known:** | | | | | | | | | | |
| **Was knowledge of the allocated intervention by outcome assessors adequately prevented?** | | | **Y** |  | **N** | |  | **Unclear** |  |  |
| **INCOMPLETE OUTCOME DATA** | | | | | | | | | | |
| **Description of the completeness of outcome data and reporting of attrition and exclusions:** | | | | | | | | | | |
| **Was the amount of incomplete outcome data (attrition and exclusions) low and was reporting adequate?** | | | **Y** |  | **N** | |  | **Unclear** |  |  |
| **SELECTIVE OUTCOME REPORTING** | | | | | | | | | | |
| **Description:** | | | | | | | | | | |
| **Are reports of the study free of suggestion of selective outcome reporting?** | | | **Y** |  | **N** | |  | **Unclear** |  |  |
| **OTHER SOURCES OF BIAS** | | | | | | | | | | |
| **Description:** | | | | | | | | | | |
| **Is the study free from other sources of bias?** | | | **Y** |  | **N** |  | | **Unclear** |  |  |
